# Effect of *Hyssopus officinalis* L. on inhibiting airway inflammation and immune regulation in a chronic asthmatic mouse model

**DOI:** 10.3892/etm.2014.1978

**Published:** 2014-09-18

**Authors:** XIAOJUAN MA, XIUMIN MA, ZHIXING MA, JING WANG, ZHAN SUN, WENYAN YU, FENGSEN LI, JIANBING DING

**Affiliations:** 1 Xinjiang National Clinical Research Base of Traditional Chinese Medicine, Xinjiang Medical University, Ürümqi, Xinjiang 830011, P.R. China; 2 Department of Immunology, College of Basic Medicine, Ürümqi, Xinjiang 830011, P.R. China; 3 Department of Clinical Laboratory, First Affiliated Hospital of Xinjiang Medical University, Ürümqi, Xinjiang 830054, P.R. China; 4 Ürümqi General Hospital of Lanzhou Military Area Command, Ürümqi, Xinjiang 830000, P.R. China

**Keywords:** chronic asthma, *Hyssopus officinalis* L., airway inflammation, immune regulation

## Abstract

The Uygur herb, *Hyssopus officinalis* L.*,* has been demonstrated to affect the levels of a number of cytokines in asthmatic mice, including interleukin-4, -6 and -17 and interferon-γ. In the present study, the effect of *Hyssopus officinalis* L. on airway immune regulation and airway inflammation was investigated in a mouse model of chronic asthma. A total of 32 BALB/c mice were randomly divided into four groups, which included the normal, chronic asthmatic, dexamethasone treatment and *Hyssopus officinalis* L.treatment groups. Mice were sensitized and challenged with ovalbumin to establish an asthma model and the ratio of eosinophils (EOS) in the bronchoalveolar lavage fluid (BALF) was determined. In addition, the levels of immunoglobulin (Ig)E and IgG were detected using an enzyme-linked immunosorbent assay. The degree of airway mucus secretion was observed using the periodic acid-Schiff stain method. The results demonstrated that the ratio of EOS in the BALF and the level of serum IgE in the chronic asthmatic and dexamethasone treatment groups increased, while the level of serum IgG decreased, when compared with the normal group. In addition, excessive secretion of airway mucus was observed in these two groups. However, the EOS ratio in the BALF and the levels of serum IgE and IgG in the *Hyssopus officinalis* L. treatment group were similar to the results observed in the normal group. In conclusion, *Hyssopus officinalis* L. not only plays an anti-inflammatory role by inhibiting the invasion of EOS and decreasing the levels of IgE, but also affects immune regulation.

## Introduction

Bronchial asthma is a common clinical disease. An epidemiological study found that the rates of morbidity, mortality and prevalence of bronchial asthma are increasing worldwide. In China, the incidence of bronchial asthma has doubled over the past decade. The World Health Organization defines bronchial asthma as a chronic airway inflammation that is caused by various inflammatory cells, including eosinophils (EOS), mast cells and T lymphocytes (1–3).

Chronic airway inflammation is characterized by the infiltration of EOS, an increase in serum immunoglobulin (Ig)E and excessive secretion of airway mucus (4–6), which result in airway structural changes and may even develop into refractory asthma or severe asthma (7). *Hyssopus officinalis* L., an Uygur medicine, is a perennial herb of Labiatae. The herb has been shown to relieve coughing and asthma; however, the underlying mechanisms are yet to be elucidated. Previous studies have shown that *Hyssopus officinalis* L. plays an anti-inflammatory role by regulating the secretion of interleukin (IL)-4, IL-17 and interferon-γ (IFN-γ), as well as regulating the imbalance between Th1/Th2 cytokines (8–10). However, the role of *Hyssopus officinalis* L. in the regulation of immune function has not yet been investigated. Therefore, the aim of the present study was to investigate the effect of *Hyssopus officinalis* L. on immunity in a mouse model of chronic asthma. In addition, the study investigated whether *Hyssopus officinalis* L. was able to increase immune reactions and thus, may be used to improve and perfect the mechanisms underlying the treatment of asthma.

## Materials and methods

### Extract preparation

Crude herbs (200 g) were extracted using 6,000 ml water. The solvents in the extracts were removed via rotary evaporation under reduced pressure (EYELA-digital water bath SB-100; Eyela, Tokyo, Japan). The aqueous extract was then freeze-dried and stored at 4°C. The aqueous extract was diluted with saline water prior to use. In a previous study, the toxicology and dosage were assessed (11).

### Animals and model establishment

A total of 32 female BALB/c mice were purchased from the Animal Experiment Center at Xinjiang Medical University (Ürümqi, China). The mice were housed in microisolator cages and received food and water. The laboratory temperature was maintained at 24±1°C, and the relative humidity was maintained between 40 and 80%. All the experimental protocols were approved by the regional Animal Ethics Committee of Xinjiang Medical University.

The BALB/c mice (age, 6–8 weeks) were randomly divided into four groups, which included the normal, chronic asthma, dexamethasone and *Hyssopus officinalis* L. groups. The chronic asthma, dexamethasone and *Hyssopus officinalis* L. groups were administered an intraperitoneal injection of 0.2 ml sensitizing agent, which contained 100 μg ovalbumin (OVA; Sigma-Aldrich, St. Louis, MO, USA) and 1 mg aluminum hydroxide gel, on days 1 and 15. In addition, initiating on day 22, the mice were administered 1% OVA for 30 min three times a week for eight weeks. The normal group were treated with phosphate-buffered saline (PBS) instead of OVA (12). Drugs were chronically administered to the animals 1 h prior to the challenges [0.005 mg/10 g body weight dexamethasone (TianYaoYaoYe Co., Ltd. Hubei, China); 0.04 g/10 g body weight *Hyssopus officinalis* L. (Pharmacy of Uyghur Hospital, Xinjiang, China)], once per day for eight weeks. Animals were euthanized following the last challenge and samples were collected.

### Bronchoalveolar lavage fluid (BALF) and serum harvesting

The chest was opened and the bronchus principalis dexter was ligated in the bifurcation. BALF was collected using 0.4 ml PBS, three times in total according to a previously described method (13,14,15). The number of neutrophils was determined using the CytoSpin™ centrifuge (Thermo Fisher Scientific, Waltham, MA, USA).

The levels of serum IgE and IgG were analyzed using an ELISA, measuring the change of absorbance at 450 nm (Bei Lai Yin Biological Technology Co., Ltd., Wuhan, China).

### Periodic acid-Schiff (PAS) staining and analysis

Lung tissue samples were stained with PAS (Jiancheng Bioengineering Institute of Nanjing, Nanjing, China) and analyzed using an optical microscope (Olympus CX22, Olympus Tokyo, Japan). A minimum of ten airways were observed in each section. The PAS positively-stained cells were counted and scored using previously described methods (16,17,18). The scoring method used was as follows: 0, <5%; l, 5–25%; 2, 25–50%; 3, 50–5%; and 4, >75% PAS positively-stained cells.

### Statistical analysis

SPSS 17.0 statistical software (SPSS, Inc., Chicago, IL, USA) was used for statistical analysis. Data are presented as the mean ± standard error of the mean. Comparisons between the groups were performed using analysis of variance followed by the Dunnett’s test, where P<0.05 was considered to indicate a statistically significant difference.

## Results

### Behavioral alterations

Following the challenge with OVA, the mice presented with a number of symptoms, including anxiety, nose scratching, coughing, inspiratory dyspnea, shortness of breath, retardation, piloerection and cyanosis. The activity decreased following continuous challenges. However, the symptoms were relieved in the dexamethasone and *Hyssopus officinalis* L. groups.

### Ratio of EOS in the BALF

**Figure 1:**
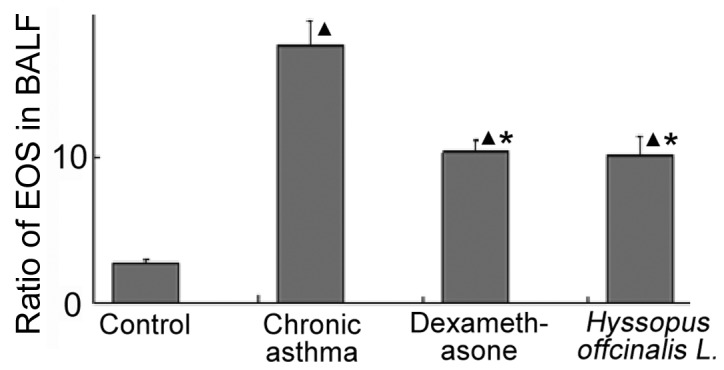
Percentage of EOS to white blood cells in the BALF. Data are presented as the mean ± standard error of the mean. ^▲^P<0.05, vs. normal group; ^▲^P<0.05, vs. chronic asthma group. BALF, bronchoalveolar lavage fluid; EOS, eosinophils

Fig. 1 shows that the percentage of EOS in the chronic asthma group was higher compared with the other groups. Furthermore, the ratio of EOS in the dexamethasone group was similar to the ratio observed in the *Hyssopus officinalis* L. group (10.27±0.85 and 10.08±1.29, respectively).

### Level of IgE and IgG in the serum

**Figure 2:**
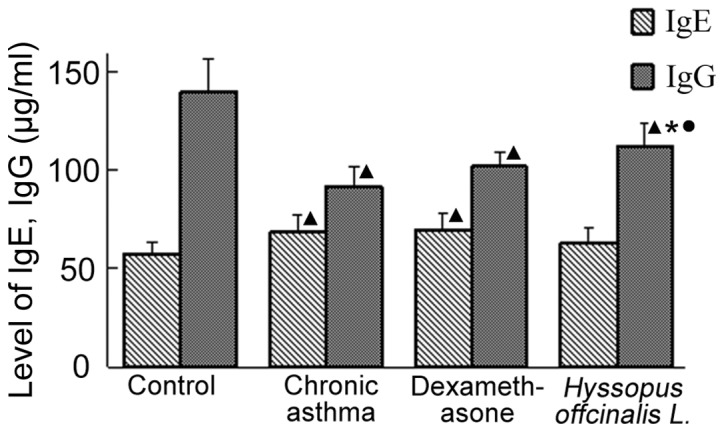
Comparison of the serum IgE and IgG concentration among the groups. Data are presented as the mean ± standard error of the mean. ^▲^P<0.05, vs. normal group; ^▲^P<0.05, vs. chronic asthma group; ^●^P<0.05, vs. dexamethasone group. Ig, immunoglobulin.

As shown in Fig. 2, the level of IgE in the *Hyssopus officinalis* L. group were similar to the levels observed in the normal group. In addition, the level of IgG in the chronic asthma and dexamethasone groups were lower compared with the normal group; however, the concentration of IgG in the *Hyssopus officinalis L.* group was higher compared with the chronic asthma and dexamethasone groups.

### Secretion of mucus in the airway

**Figure 3:**
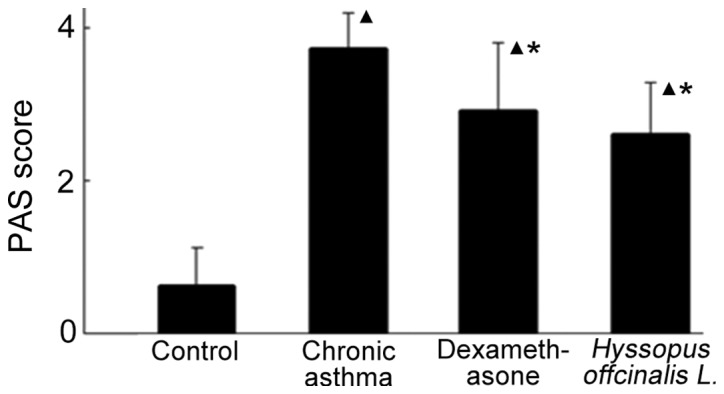
Comparison of airway mucus secretion among the groups. Data are presented as the mean ± standard error of the mean. ^▲^P<0.05, vs. normal group; ^▲^P<0.05, vs. chronic asthma group. PAS, periodic acid-Schiff.

**Figure 4:**
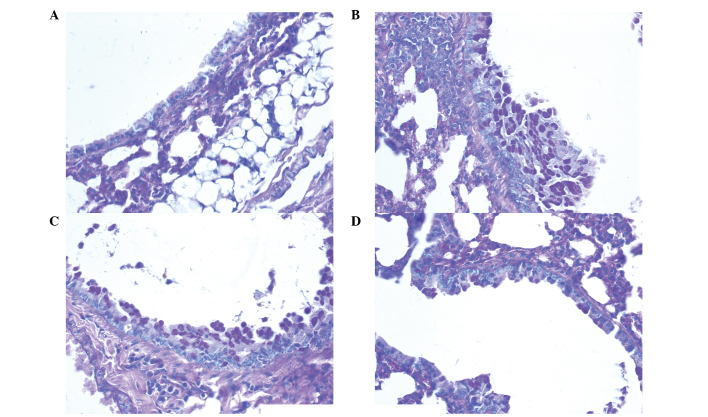
Lung tissue staining by PAS was used to observe the secretion of airway mucus in the (A) normal, (B) chronic asthma, (C) dexamethasone and (D) *Hyssopus offcinalis* L. groups (magnification, ×40). Analysis shows the neutral mucus as purple in the alveolar space and the nucleus as violet. PAS, periodic acid-Schiff.

The secretion of airway mucus in the chronic asthma group was aggravated (Figs. 3 and 4). In addition, the secretion of mucus increased in the dexamethasone and *Hyssopus officinalis* L. groups; however, the levels were highest in the chronic asthma group.

## Discussion

Asthma is a type I allergic disease, and IgE has an important role in the development of asthma (19). Previous studies have demonstrated that there are various levels of inflammatory responses in patients who suffer from asthma, and allergic airway inflammation with increasing EOS is the main pathological feature of asthma (20,21). Numerous studies have identified a correlation between the infiltrate level of EOS and the severity of airway inflammation (22–24). The variety of cell types present in BALF may reflect the degree of inflammation in the peripheral airways, which is also the main factor causing bronchial hyperresponsiveness in asthma (25). Therefore, airway inflammation is the basic condition of reversible airway inhibition, as well as non-special hyperresponsiveness of the bronchus. In the present study, the percentage of EOS in the BALF and the levels of IgE in the chronic asthma group were higher compared with the normal group (P<0.05). PAS staining revealed that mucus secretion was elevated in the chronic asthma group. Furthermore, the level of IgG was observed to decrease in the chronic asthma group. These results indicated that the immune reaction in individuals suffering chronic asthma is restrained.

Hormonal nebulizer inhalation has become a common clinical treatment for non-special airway inflammation; however, this suppresses the immune system. Due to the geography and climate of the Autonomous region of Xinjiang, the incidence of asthma is increasing. There are a number of effective strategies and medicines for treating asthma in Uygur medicine. *Hyssopus officinalis* L. is an Uygur medicine used for the treatment of a number of conditions, including asthma, coughing, fever and rheumatism (26). In a number of previous studies, *Hyssopus officinalis* L. has been demonstrated to affect the expression of a number of cytokines (8–10). In the present study, mice treated with *Hyssopus officinalis* L. and dexamethasone were shown to have decreased levels of EOS in the BALF when compared with mice in the chronic asthma group (10.08±1.29, 10.27±0.85 and 17.56±1.71, respectively; P<0.05). In addition, the level of IgE and the secretion of airway mucus were reduced. Mice treated with *Hyssopus officinalis* L. were also shown to have higher levels of IgG when compared with the dexamethasone and asthma groups (111.71±11.79, 101.69±7.02 and 91.27±10.69, respectively; P<0.05). These observations indicate that *Hyssopus officinalis* L. may exhibit an anti-inflammatory effect by inhibiting the infiltration of EOS and reducing the level of IgE in the lung tissue; thus, regulating immunity.

In conclusion, the results of the present study demonstrate the effect of *Hyssopus officinalis* L. on chronic asthma, and may provide a novel therapeutic strategy for the treatment of chronic asthma. However, further investigation is required to determine the specific mechanism.
